# Antibiotic Rotation for Febrile Neutropenic Patients with Hematological Malignancies: Clinical Significance of Antibiotic Heterogeneity

**DOI:** 10.1371/journal.pone.0054190

**Published:** 2013-01-23

**Authors:** Yong Chong, Shinji Shimoda, Hiroko Yakushiji, Yoshikiyo Ito, Toshihiro Miyamoto, Tomohiko Kamimura, Nobuyuki Shimono, Koichi Akashi

**Affiliations:** 1 Department of Medicine and Biosystemic Sciences, Kyushu University Graduate School of Medicine, Fukuoka, Japan; 2 Department of Clinical Laboratory, Hara-Sanshin Hospital, Fukuoka, Japan; 3 Department of Blood and Marrow Transplantation, Hara-Sanshin Hospital, Fukuoka, Japan; 4 Center for the Study of Global Infection, Kyushu University Hospital, Fukuoka, Japan; Fundacion Huesped, Argentina

## Abstract

**Background:**

Our unit adopted the single administration of cefepime as the initial treatment for febrile episodes in neutropenic patients with hematological malignancies. However, recently, cefepime-resistant gram-negative bacteremia, including those with extended-spectrum β-lactamase (ESBL)-producers, was frequently observed in these patients. Therefore, we instituted a rotation of primary antibiotics for febrile neutropenic patients in an attempt to control antibiotic resistance.

**Methods:**

This prospective trial was performed from August 2008 through March 2011 at our unit. After a pre-intervention period, in which cefepime was used as the initial agent for febrile neutropenia, 4 primary antibiotics, namely, piperacillin-tazobactam, ciprofloxacin, meropenem, and cefepime, were rotated at 1-month intervals over 20 months. Blood and surveillance cultures were conducted for febrile episodes, in order to assess the etiology, the resistance pattern (particularly to cefepime), and the prognosis.

**Results:**

In this trial, 219 patients were registered. A 65.9% reduction in the use of cefepime occurred after the antibiotic rotation. In the surveillance stool cultures, the detection rate of cefepime-resistant gram-negative isolates, of which ESBL-producers were predominant, declined significantly after the intervention (8.5 vs 0.9 episodes per 1000 patient days before and after intervention respectively, *P*<0.01). Interestingly, ESBL-related bacteremia was not detected after the initiation of the trial (1.7 vs 0.0 episodes per 1000 patient days before and after intervention respectively, *P*<0.01). Infection-related mortality was comparable between the 2 periods.

**Conclusions:**

We implemented a monthly rotation of primary antibiotics for febrile neutropenic patients. An antibiotic heterogeneity strategy, mainly performed as a cycling regimen, would be useful for controlling antimicrobial resistance among patients treated for febrile neutropenia.

## Introduction

Febrile neutropenia is one of the most serious adverse events in patients with hematological malignancies and chemotherapy. A prompt initiation of empirical antibiotic therapy is favorable for patients with febrile neutropenia, regardless of the detection of bacteremia. Based on guidelines [1], [2], our unit had adopted single administration of cefepime, a fourth-generation cephalosporin, as the initial treatment for febrile episodes in neutropenic patients. However, recently, cefepime-resistant gram-negative bacteremia, including those with extended-spectrum β-lactamase (ESBL)-producers, was frequently seen in these patients [3]. ESBL-producing bacteria have been frequently detected not only in our hematology unit, but in all the units at our hospital. Therefore, we considered rotating primary antibiotics for the treatment of febrile neutropenic episodes as a measure to suppress the antibiotic resistance seen in our unit.

Most antibiotic rotation trials, known as cycling therapies, have been conducted in adult intensive-care units (ICUs), where infectious agents are exposed to heavy antimicrobial pressure. Many cycling therapies implemented in clinical settings have been reported to date. Most of these trials were conducted in order to reduce the selective pressure of antibiotic-resistant gram-negative bacteria, which was a major concern for patient prognosis in an ICU setting. The efficacy of antibiotic cycling on the recovery of antimicrobial susceptibility and patient mortality is controversial. Several trials have successfully reported a recovery of susceptibility to specific antibiotics or a reduction in detection rates of multidrug-resistant bacteria [4]–[9]. Moreover, some studies have shown a significant decrease in mortality as a result of the trial [4], [5]. However, other trials did not report recovery of susceptibility to a given antibiotic after antibiotic rotation [10], [11]. Due to the contradictory results of several trials, no conclusion has been reached about the benefits of antibiotic rotation on controlling antimicrobial resistance [12]–[14].

Cycling therapies have been instituted as the primary treatment for febrile neutropenic patients in hematological units [15]–[19]. Most of these attempts were focused on controlling antibiotic resistance among gram-negative bacteria [16]–[19]. From these studies, no conclusive findings have been obtained on the efficacy of antibiotic rotation strategies in patients with febrile neutropenia. Reasons for this include the following: (1) most studies did not clearly show a significant decrease in the frequency of antibiotic-resistant bacteria after intervention, and (2) some studies implemented the antibiotic cycling program in patients receiving fluoroquinolone prophylaxis, which is known to dramatically change the etiology and resistance pattern of infectious agents recovered from patients with febrile neutropenia [20], [21]. Therefore, precise evaluation of the benefits of cycling therapy on suppressing antibiotic resistance in a program of antibiotic prophylaxis is difficult.

For the abovementioned reasons, the efficacy of antibiotic cycling in reducing antibiotic resistance among patients with hematological malignancies, as well as those in ICUs, has not been determined. However, patients with febrile neutropenia, which often develops in those with hematological malignancies, are expected to apply for antibiotic rotation strategy in controlling antibiotic resistance. Febrile neutropenia is a unique infectious disease. Broad-spectrum antibiotics are often administered to febrile neutropenic patients for long periods, without the identification of the causative bacteria, until the neutrophil count recovers. A few classes of antimicrobial agents are recommended as the first line of treatment for febrile neutropenic patients. As a result, overuse of some antibiotics in a small population increases the risk of selecting antibiotic-resistant bacteria. Therefore, if cycling therapy reduces the selection of antibiotic-resistant bacteria, this therapeutic regimen may be beneficial in patients with febrile neutropenia following hematological malignancies.

We prospectively conducted a systematic cycling program for initial use of antibiotics in febrile neutropenic patients in our unit. After a prospective observational period of cefepime administration, 4 antibiotics were rotated monthly over 20 months without quinolone prophylaxis. We observed a significant decrease in the rates of antibiotic resistance. This is the first study to show a significant reduction in antibiotic resistance in a hematology and febrile neutropenia setting after use of antibiotic cycling. Further discussion on the benefits and difficulties of introduction of antibiotic rotation strategies is warranted in hematological settings.

## Methods

### Patients and Ethics Statement

This study was conducted in a single hematological unit with 37-beds at the Hara-Sanshin Hospital. The study was prospectively conducted from August 2008 to March 2011. The protocol was approved, through the ethics review process, by the Institutional Review Board of the Hara-Sanshin Hospital. Written informed consent was obtained from all registered patients before the study protocol was implemented, in order to publish these case details. Infection control measures, including hand-washing promotion and isolation procedures, were maintained at the same intensity before and after the initiation of the study.

### Enrollment

The study protocol was applied for patients with febrile neutropenia who had provided informed consent. The febrile neutropenia defined in this study needs to fulfill both of fever and neutropenia as follows: (1) fever was defined as a single axillary temperature >38.0°C or a temperature of >37.5°C sustained over a 1-h period, and (2) neutropenia was defined as an absolute neutrophil count (ANC) of <1,000 cells/mm^3^ or an ANC that was expected to decrease to <1,000 cells/mm^3^ during the following 48 h. Exclusion criteria included treatment for allogeneic hematopoietic stem cell transplantation (HSCT), evidence of hepatic and/or renal dysfunctions (defined as a serum transaminase level of more than 3 times the upper limit of the normal range or as a serum creatinine level of more than 1.5 times the upper limit of the normal range), and a history of hypersensitivity to β-lactam antimicrobial agents. Patients receiving an allogeneic HSCT were excluded from the trial, because a confirmative diagnosis of febrile neutropenia is often difficult to make due to the presence of other causative factors such as graft-versus-host disease and engraftment syndrome. The routine use of fluoroquinolone prophylaxis for neutropenic patients was discontinued in January 2006 at our unit.

### Treatment protocol

From August 2008 to July 2009, cefepime was used as the initial agent for treating febrile neutropenia in the registered patients. Next, 4 primary antibiotics, namely, piperacillin-tazobactam, ciprofloxacin, meropenem, and cefepime, were rotated at 1-month intervals over 20 months from August 2009 to March 2011. Ciprofloxacin was adopted as a rotated agent in addition to the 3 other antibiotics recommended for febrile neutropenia [22]. Most cycling studies conducted in ICU settings have used fluoroquinolones as one of the rotated agents because quinolones are highly effective against gram-negative bacteria. In addition, no prophylaxis with quinolones was performed during the study. There is no recommendation for the optimal duration of antibiotic cycling. Although many studies have rotated antibiotics every 3 months, significant evidence on the benefits of cycling therapy has not been obtained from this duration. Some groups have reported that monthly rotation has a positive effect on suppressing antibiotic resistance [7], [8]. Theoretically, a shorter interval of antibiotic cycling is expected to have a lower selection pressure on resistant bacteria [23]. Considering the results to date, monthly cycling was adopted in this study.

The Standard dosage of antibiotics rotated in this study was piperacillin-tazobactam 4.5 g intravenous (i.v.) every 8 h, ciprofloxacin 0.3 g i.v. every 12 h, meropenem 0.5–1 g i.v. every 8 h, and cefepime 2 g i.v. every 12 h. Meropenem (2 g/day) was administered in unequally divided doses. A daily dose of ciprofloxacin, meropenem and cefepime was the maximum dosage approved for them in Japan. The administration of antibiotics was continued until recovery of neutrophil counts and/or resolution of infection. When causative bacteria were isolated from a sample of culture, antimicrobial therapy was adjusted according to the antibiotic resistance pattern obtained. The degrees of consumption of rotated antibiotics were compared before and after the intervention, using the defined daily dose (DDD) per 1000 patient days as an index for the evaluation. The DDD of each antibiotic is defined by the World Health Organization.

### Microbiology

When the registered neutropenic patients had fever defined as above, blood and stool samples were collected. Several samples were obtained from the same patient and treated as independent results. If multiple organisms were detected from a single sample, they were counted and analyzed as independent isolates. An automated blood culture system (BACTEC) was used for each test. Stool samples were cultured, chiefly using 5% Sheep Blood Agar medium (BD) and CHROMagar Candida medium (BD). The species were identified using the Vitek system (bioMerieux Japan Ltd., Tokyo, Japan). Antibiotic susceptibilities were determined by the breakpoints standardized by the Clinical and Laboratory Standards Institute (CLSI; formerly the NCCLS) [24]. The screening and confirmation tests for ESBL and metallo-β-lactamase were conducted according to the recommendation of the CLSI [24]. In addition, β-lactamase producers were confirmed using a Cica β test I/MBL kit (Kanto Chemical Co. Ltd., Tokyo, Japan). *Clostridium difficile* toxin A and B were examined in stool samples, using a TOX A/B QUIK CHEK kit (Nissui Pharmaceutical Co. Ltd., Tokyo, Japan).

### Statistical analysis

In the comparisons between patients receiving pre-intervention and those receiving the antibiotic rotation trial, categorical variables were analyzed using a Fisher's exact test, and continuous variables were compared using the Mann-Whitney *U* test. *P*<0.05 was considered to be statistically significant. All statistical calculations were performed using the SAS software (SAS Institute, Inc., Cary, NC, USA).

## Results

### Characteristics of patients registered for antibiotic rotation therapy

In this study, 219 patients were enrolled. Seventy-one patients were registered with 2350 patient days before the antibiotic rotation. After the initiation of cycling therapy, 148 patients were recorded, accounting for 4592 patient days. Some patients were enrolled multiple times for sequential chemotherapies. The basic characteristics of the registered patients were found to be different before and after the rotation, as shown in Table 1. The multinational association for supportive care in cancer (MASCC) score was comparable between the 2 groups, suggesting that both groups were at a similar risk for serious complications and death [22].

**Table 1:** Characteristics of patients before and after intervention.

Variable	Before, n = 71	After, n = 148	*P*
Age, mean years±SD (range)	62.0±10.6 (40–82)	59.0±17.0 (17–87)	0.86
Male sex	58 (81.7)	95 (64.2)	<0.05
Malignant disease			
Leukemia	27 (38.0)	78 (52.7)	<0.05
Lymphoma	20 (28.2)	46 (31.1)	0.75
MDS	7 (9.9)	9 (6.1)	0.40
Multiple myeloma	17 (23.9)	7 (4.7)	<0.01
Other	0 (0.0)	8 (5.4)	0.06
Therapy for hematological disorders			
Chemotherapy	61 (85.9)	136 (91.9)	0.23
Autologous HSCT	9 (12.7)	8 (5.4)	0.10
Therapy for infectious diseases			
Use of G-CSF	41 (57.7)	71 (48.0)	0.20
Use of Antimycotic drugs	54 (76.1)	84 (56.8)	<0.01
Intravenous hyperalimentation catheter	49 (69.0)	115 (77.7)	0.19
Neutrophil cell count (/uL), mean	299.5±326.0 (0–968)	155.7±258.2(0–1270)	<0.05
number ± SD (range)			
MASCC score ± SD (range)	21.3±1.8 (16–24)	21.4±1.4 (17–23)	0.94

*P*-value shows statistical comparison for each variable between patients receiving pre-intervention and those receiving intervention.

MDS, myelodysplastic syndromes;HSCT, hematopoietic stem cell transplantation.

G-CSF, granulocyte colony-stimulating factor; MASCC, multinational association for supportive care in cancer.

### Effects of antibiotic rotation on the etiology of bacteremic and colonized isolates

Before the initiation of the cycling therapy, 23 bacterial strains were isolated from the blood cultures of the patients, with a total of 71 episodes of febrile neutropenia (Table 2). During cycling therapy, 33 bacteria were recovered from blood culture isolates associated with 148 episodes of febrile neutropenia (Table 2). After the antibiotic rotation trial, the incidence of gram-negative isolates decreased, but this decrease was not statistically significant. Gram-positive organisms were detected comparatively in both the periods. Methicillin-resistant *Staphylococcus aureus* (MRSA) was first detected after the intervention. The incidence of *Enterococcus* species was similar in both periods. Vancomycin-resistant enterococci (VRE) were not isolated in either of the 2 periods.

**Table 2:** Effect of antibiotic rotation on the etiology of bacteremic isolates.

	Before cycling		After cycling		
Organism	No. of isolates	Rate	No. of isolates	Rate	*P*
Gram-negative					
*Escherichia coli*	6	2.6	7	1.5	0.38
*Pseudomonas aeruginosa*	4	1.7	3	0.7	0.24
*Klebsiella pneumoniae*	3	1.3	3	0.7	0.41
Other	0	0.0	1	0.2	1.00
Gram-negative, total	13	5.5	14	3.1	0.15
Gram-positive					
*Staphylococcus* species, total	7	3.0	14	3.1	1.00
Coagulase negative staphylococci	6	2.6	11	2.4	1.00
*Staphylococcus aureus*	1	0.4	1	0.2	1.00
*Enterococcus* species, total	2	0.9	2	0.4	0.61
*Enterococcus faecium*	2	0.9	1	0.2	0.27
*Enterococcus faecalis*	0	0.0	1	0.2	1.00
*Streptococcus* species, total	1	0.4	3	0.7	1.00
*Viridans Group streptococci*	1	0.4	3	0.7	1.00
Gram-positive, total	10	4.3	19	4.1	1.00
Other	0	0.0	0	0.0	1.00
Isolates, total	23	9.8	33	7.2	0.26

Rate indicates number of positive cultures per 1000 patient days.

*P*-value shows statistical comparison for each variable between patients receiving pre-intervention and those receiving intervention.

Acquisition of VRE, as a causative and/or colonized organism, has been reported after antibiotic rotation in both ICUs and hematological units [17], [18], [25]. This implies that antibiotic cycling for preventing multidrug-resistant gram-negative infections affects the entire bacterial flora of patients receiving the therapy. Therefore, stool cultures were performed not only to examine the colonization of multidrug-resistant gram-negative bacteria, but also to observe changes in the enteric bacterial flora of the patients after antibiotic rotation. As shown in Table 3, the detection rate of *Escherichia coli* isolates significantly decreased after antibiotic rotation. The incidence of gram-positive isolates was similar in both periods. MRSA was recovered from enteric cultures as well as blood cultures after antibiotic rotation. The incidence of a whole of *Enterococcus* species was comparable in both periods; however, the number of *Enterococcus faecium* isolated significantly increased after the antibiotic rotation. The colonization of VRE was not seen in either of the 2 periods. *Candida* species were detected more frequently after the cycling therapy.

**Table 3:** Effect of antibiotic rotation on the etiology of stool isolates.

	Before cycling		After cycling		
Organism	No. of isolates	Rate	No. of isolates	Rate	*P*
Gram-negative					
*Escherichia coli*	45	19.2	53	11.5	<0.05
*Klebsiella pneumoniae*	19	8.1	52	11.3	0.26
*Pseudomonas aeruginosa*	5	2.1	4	0.9	0.18
Other	20	8.5	48	10.5	0.52
Gram-negative, total	89	37.9	157	34.2	0.34
Gram-positive					
*Staphylococcus* species, total	46	19.6	76	16.6	0.39
Coagulase negative staphylococci	46	19.6	74	16.1	0.33
*Staphylococcus aureus*	0	0.0	2	0.4	0.55
*Enterococcus* species, total	66	28.1	129	28.1	1.00
*Enterococcus faecium*	7	3.0	33	7.2	<0.05
*Enterococcus faecalis*	29	12.3	55	12.0	0.91
*Streptococcus* species, total	17	7.2	34	7.4	1.00
*Viridans Group streptococci*	17	7.2	26	5.7	0.42
Other	7	3.0	26	5.7	0.14
Gram-positive, total	136	57.9	265	57.7	1.00
Other	2	0.9	16	3.5	<0.05
Isolates, total	227	96.6	438	95.4	0.86

Rate indicates number of positive cultures per 1000 patient days.

*P*-value shows statistical comparison for each variable between patients receiving pre-intervention and those receiving intervention.

### Antimicrobial susceptibilities of gram-negative isolates from patients with bacteremia and colonization

As the purpose of this study was to examine the susceptibility pattern of multidrug-resistant gram-negative organisms after the intervention, we analyzed the antibiotic sensitivity of gram-negative isolates, rather than gram-positive isolates. The incidence of bacteremic gram-negative isolates resistant to the cycled antibiotics was compared between the 2 periods (Table 4). In the period before the cycling therapy, 6 of the 13 gram-negative isolates were resistant to cefepime. Approximately 70% of the cefepime-resistant isolates were found to be ESBL-producing strains. For the duration of the cycling therapy, 1 of 14 gram-negative isolates was resistant to cefepime and this isolate was identified as *P. aeruginosa* (Table 4). ESBL-producing organisms were not detected during the cycling therapy. The detection rates of cefepime-resistant and ESBL-producing isolates after the antibiotic rotation were significantly lower than those before the rotation.

**Table 4:** Antibiotic resistance of bacteremic isolates before and after intervention.

			CFPM-resistant				
		Total No.	Rate (n) of isolates	Rate (n) of isolates	Rate (n) of isolates	Rate (n) of isolates	Rate (n) of isolates
Organism		of isolates	resistant to CFPM	producing ESBL	resistant to MEPM	resistant to PIPC/TAZ	resistant to CPFX
Gram-negative, total	Before	13	2.6 (6)^a^	1.7 (4)^b^	0.0 (0)	0.0 (0)	2.1 (5)^c^
	After	14	0.2 (1)^a^	0.0 (0)^b^	0.0 (0)	0.0 (0)	0.4 (2)^c^

Rate indicates number of positive cultures per 1000 patient days.

a,b,c *P*-value shows statistical comparison for each variable between patients receiving pre-intervention and those receiving intervention.

a *p* = 0.007,

b *p* = 0.013,

c *p* = 0.048.

CFPM, cefepime; ESBL, extended-spectrum β-lactamase; MEPM, meropenem; PIPC/TAZ, piperacillin-tazobactam; CPFX, ciprofloxacin.

The frequencies of gram-negative isolates resistant to the cycled antibiotics recovered from stool cultures both before and after antibiotic rotation are shown in Table 5. For the period before antibiotic rotation, 20 of 89 gram-negative isolates, including 15 of 45 *E. coli* isolates and 3 of 19 *K. pneumoniae*, were resistant to cefepime and most of them were ESBL-producers. Other gram-negative isolates resistant to cefepime were *Citrobacter koseri*, which produce ESBL. For the period of antibiotic rotation, 4 of the 157 gram-negative isolates were ESBL-producing *E. coli* isolates that were cefepime-resistant. No resistance to cefepime was detected from organisms other than *E. coli*. The rates of cefepime-resistant and ESBL-producing gram-negative isolates, including *E. coli* and *K. pneumoniae* strains, obtained after antibiotic cycling were significantly lower than those obtained before the cycling.

**Table 5:** Antibiotic resistance of stool isolates before and after intervention.

			CFPM-resistant				
		Total No.	Rate (n) of isolates	Rate (n) of isolates	Rate (n) of isolates	Rate (n) of isolates	Rate (n) of isolates
Organism		of isolates	resistant to CFPM	producing ESBL	resistant to MEPM	resistant to PIPC/TAZ	resistant to CPFX
Gram-negative, total	Before	89	8.5 (20)^a^	7.7 (18)^b^	0.0 (0)	0.0 (0)	14.9 (35)^c^
	After	157	0.9 (4)^a^	0.9 (4)^b^	0.0 (0)	0.0 (0)	5.7 (26)^c^
*Escherichia coli*	Before	45	6.4 (15)^d^	5.5 (13)^e^	0.0 (0)	0.0 (0)	14.0 (33)^f^
	After	53	0.9 (4)^d^	0.9 (4)^e^	0.0 (0)	0.0 (0)	5.0 (23)^f^
*Klebsiella pneumoniae*	Before	19	1.3 (3)^g^	1.3 (3)^h^	0.0 (0)	0.0 (0)	0.0 (0)
	After	52	0.0 (0)^g^	0.0 (0)^h^	0.0 (0)	0.0 (0)	0.7 (3)
*Pseudomonas aeruginosa*	Before	5	0.0 (0)	0.0 (0)	0.0 (0)	0.0 (0)	0.0 (0)
	After	4	0.0 (0)	0.0 (0)	0.0 (0)	0.0 (0)	0.0 (0)
Other	Before	20	0.9 (2)	0.9 (2)	0.0 (0)	0.0 (0)	0.9 (2)
	After	48	0.0 (0)	0.0 (0)	0.0 (0)	0.0 (0)	0.0 (0)

Rate indicates number of positive cultures per 1000 patient days.

a,b,c,d,e,f,g,h *P*-value shows statistical comparison for each variable between patients receiving pre-intervention and those receiving intervention.

a *p*<0.0001,

b *p*<0.0001,

c *p*<0.01.

d *p*<0.0001,

e *p*<0.001,

f *p*<0.001,

g *p*<0.05,

h *p*<0.05.

CFPM, cefepime; ESBL, extended-spectrum β-lactamase; MEPM, meropenem; PIPC/TAZ, piperacillin-tazobactam; CPFX, ciprofloxacin.

### Antibiotic use

The dosages of the rotated antibiotics used within our unit were calculated by including not only the registered patients but also the other non-registered patients for the duration of this study. As shown in Table 6, the DDD per 1000 patient days of cefepime was 211 and 72 before and after the intervention, respectively. Thus, the unit-wide cefepime use reduced by 65.9% after the antibiotic rotation trial. The reduction rate of cefepime use remained stable throughout the period of the cycling program (Table 6).

**Table 6:** Unit-wide antibiotic use shown as defined daily dose.

	Before cycling	After cycling					
		1st-5th	1st	2nd	3rd	4th	5th
Antibiotic	Aug/08-Jul/09	Aug/09-Mar/11	Aug/09-Nov/09	Dec/09-Mar/10	Apr/10-Jul/10	Aug/10-Nov/10	Dec/10-Mar/11
CFPM	211	72	81	51	60	93	77
MEPM	98	82	89	92	80	72	79
PIPC/TAZ	149	543	472	587	406	617	632
CPFX	5	30	27	39	26	19	38

Each number is shown to defined daily dose per 1000 patient days.

CFPM, cefepime; MEPM, meropenem; PIPC/TAZ, piperacillin-tazobactam; CPFX, ciprofloxacin.

In several cases, an inadequate antibiotic therapy was administered due to antibiotic resistance. Antibiotics were then changed. During the pre-intervention period, in which cefepime was used, 6 isolates of cefepime-resistant gram-negative bacteria, comprising 4 cases of ESBL-producers and 2 cases of *P. aeruginosa*, were detected from blood culture. After the intervention trial, 1 isolate of ciprofloxacin-resistant *E. coli* was detected when ciprofloxacin was used as an initial antibiotic. An empirical change to the initial antibiotic therapy was carried out due to persistent fever; this was done without a microbiological isolation. The antibiotic change was applied to 14 cases and 38 cases, before and after the trial, respectively.

### Mortality

The antibiotic agents adopted in this study were well tolerated, regardless of the intervention. Although clinical and laboratory abnormalities, including skin rash, diarrhea, and elevation of serum transaminase level, were observed, these occurred at a frequency of less than 5% and were not severe. *C. difficile*-associated diarrhea was observed in 2 patients receiving cefepime or piperacillin-tazobactam for the duration of the trial. We recorded 2 deaths (2.8%) during the pre-cycling period and 4 (2.7%) during the cycling period (*P* = 1.0). One patient died during the trial due to possible septic shock caused by *E. coli*. No infection-related death was seen before cycling therapy.

## Discussion

In this trial, cefepime administration, as a result, dramatically declined at a unit-wide level after antibiotic rotation (Table 6). Thus, the antibiotic rotation designed to decrease the antibiotic pressure due to the dominant use of cefepime led to a unit-wide reduction in cefepime use. This means that antimicrobial use for febrile neutropenia has a huge impact on antibiotic intake throughout a hematological unit. Further to the revised guideline [22], piperacillin-tazobactam was newly recommended as a first-line agent for febrile neutropenia, in addition to cephalosporins and carbapenems. The increase in the number of the initial agents recommended for febrile neutropenia, with different antibiotic mechanisms, significantly contributes to decreasing the dominant use of a particular agent. The introduction of piperacillin-tazobactam also facilitated the initiation of this antibiotic rotation trial.

The primary purpose of this study was to investigate whether antibiotic rotation, when used as the first line of treatment for febrile neutropenia, contributes to controlling the spread of antibiotic resistance; a significant reduction in the incidence of cefepime-resistant and ESBL-producing organisms was observed after antibiotic rotation (Table 4 and 5). The dosage of cefepime used throughout our unit decreased to approximately 30% after the initiation of antibiotic rotation (Table 6). The constant use of broad-spectrum cephalosporins is known to be associated, as an independent risk factor, with the emergence of ESBL-producing bacteria [26]. In addition, the restricted use of cephalosporins was reported to reduce the detection rates of ESBL-producing organisms [27]–[30]. Interestingly, a trial of cephalosporin restriction, conducted by Rahal et al. showed a significant decrease in ESBL-producing *Klebsiella* infection and colonization within 1 year [27]. Although the period of the intervention was relatively short, the antibiotic rotation trial, followed by a decline in cephalosporin consumption, may be related to the successful control of multidrug-resistant bacteria.

**Figure 1:**
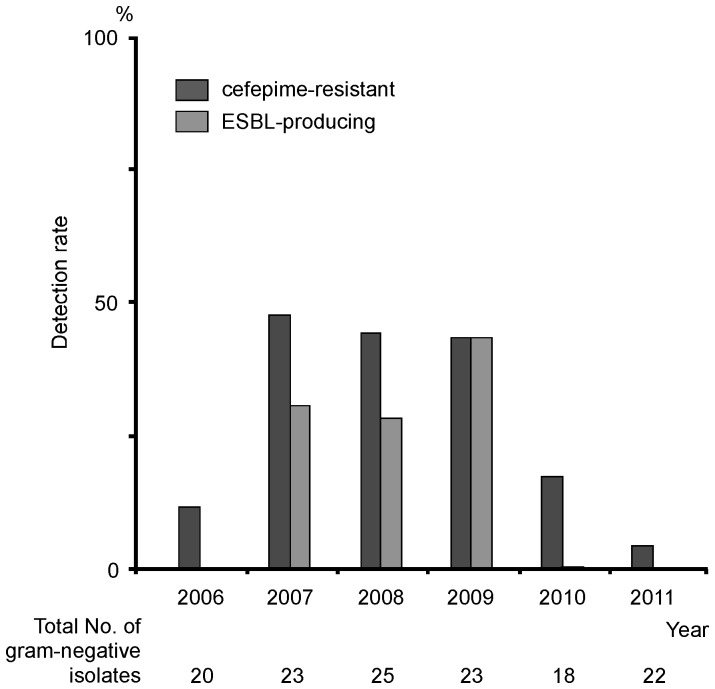
Unit-wide frequency of cefepime-resistant and extended-spectrum β-lactamase producing strains in bacteremic isolates obtained from patients with febrile neutropenia. The detection rates of cefepime-resistant and extended-spectrum β-lactamase **(**ESBL)-related bacteremia were retrospectively analyzed throughout a hematological unit at the Hara-Sanshin Hospital. The frequency (%) indicates the rate of cefepime-resistant and ESBL-producing isolates obtained from total gram-negative bacteremic isolates. The antibiotic rotation trial was conducted from August 2009 to March 2011.

There is a concern regarding the quality of this trial. A control group, to be compared to the enrolled group, was not included in our study. To split a single unit into 2 sections was needed in order to set up a definite control group. In this case, the control group included patients that were under continuous antibiotic pressure induced by the dominant use of cefepime. Finally, we abandoned preparing a control group due to concerns about feasibility. Retrospectively, we tried to analyze the detection rates of cefepime-resistant bacteremic organisms throughout our unit, including in registered and non-registered patients. As shown in Figure 1, the frequency of cefepime-resistant bacteremia, including ESBL-related bacteremia, dramatically decreased after antibiotic rotation. A unit-wide reduction in cefepime use may lead to a unit-wide decrease in the susceptibility of antibiotic resistance. We think that this finding further supports the positive impact of antibiotic rotation in controlling antimicrobial resistance.

Antibiotic rotation may affect the etiology of bacteria detected, for not only gram-negative but also gram-positive organisms. In antibiotic cycling trials conducted for febrile neutropenic patients with hematological malignancies, an increase in the incidence of *Enterococcus* sp., as an agent of infection and colonization, has been observed [16]–[18], and the emergence of VRE has especially been of concern [17], [18]. Craig et al. reported that the detection rates of vancomycin-resistant *E. faecium* dramatically increased, in the form of bacteremic isolates, after adoption of antibiotic rotation [17]. Antibiotic cycling may be related to the appearance of VRE in ICU settings as well as in hematological units [25]. In this study, the number of *Enterococcus* isolates detected from blood did not increase after antibiotic rotation; however, a stool culture showed a significant increase in *E. faecium* after antibiotic rotation (Table 2 and 3). No VRE isolates were detected in this trial. The use of antibiotics with anaerobic activity may be related to the acquisition of VRE [17], [18], [25]. In our trial, piperacillin-tazobactam and meropenem, 2 of the 4 antimicrobials adopted, are active against anaerobic bacteria. The lack of detection of VRE in our unit may be due to the epidemiology of VRE. The environment around our hospital maintains a low prevalence of VRE among the *E. faecium* isolates found here. Thus, if VRE is epidemic in a certain area, colonization pressure on VRE may be higher after a trial of antibiotic rotation. Further studies will be necessary to confirm this hypothesis.

In this trial, ciprofloxacin was used as the initial agent for the treatment of febrile neutropenia. This selection was based on ICU-oriented regimens, which focus on the control of multidrug-resistant gram-negative bacteria, such as *P. aeruginosa*. A guideline issued by the IDSA dose not recommend monotherapy with ciprofloxacin as an initial treatment for febrile neutropenia [22]. Intriguingly, *E. coli* isolates resistant only to fluoroquinolones, including ciprofloxacin and levofloxacin, were frequently detected in this study, even though the routine use of quinolone prophylaxis was avoided in febrile neutropenic patients. The epidemic spread of quinolone-resistant *E. coli* is characteristic as a local factor. Considering these results, it may be difficult, in our institution to use fluoroquinolones as an initial monotherapy for febrile neutropenia, even if the quinolones have anti-pseudomonal activity.

Whether antibiotic cycling strategies contribute to decreased mortality has not been addressed. However, some studies reported improved prognosis after antibiotic rotation [4], [5]. No studies have shown a decline in mortality in cycling trials for febrile neutropenic patients with hematological malignancies. Our study showed no difference in mortality before and after implementation of antibiotic rotation. This may be partially due to the low mortality of patients undergoing therapy before antibiotic rotation; these patients received cefepime as an initial treatment for febrile neutropenia. ESBL-producing organisms were frequently recovered from blood cultures before antibiotic rotation was performed in our unit. In most cases of patients with ESBL-producing isolates, a prompt antibiotic change to carbapenems resulted in successful treatment and good prognosis [3]. The clinical outcome of bacteremia caused by ESBL-producing organisms remains controversial [26]; however, inappropriate use of empirical antibiotics for ESBL-related bacteremia possibly results in high mortality [31]–[33]. A consistent decline in ESBL-related bacteremia may contribute to a decreased risk for fatality over a longer observation period.

We implemented an antibiotic rotation protocol conducted in our institution, in which 4 antibiotics were rotated at 1-month intervals. Multiple compliance issues with the protocol were appropriately overcome by the presence of properly educated staff, including physicians, nurses, pharmacists, and laboratory technicians. However, due to the limited feasibility of such a protocol, it will be difficult to adopt such a cycling program widely, even if antibiotic resistance could be controlled by this rotation strategy. The most important conclusion of this study is not that this cycling regimen is needed to control antimicrobial resistance, but that the maintenance of “antibiotic heterogeneity” may be effective in reducing the selection pressure on antibiotic resistance. Antibiotic heterogeneity can be achieved by antibiotic mixing, as well as antibiotic cycling [14]. Bergstrom et al theoretically estimated that antibiotic mixing may be more beneficial than antibiotic cycling [23]. In addition, a new strategy of Periodic Antibiotic Monitoring and Supervision (PAMS) has been recently proposed for controlling antimicrobial resistance [34]. If an antibiotic rotation strategy is not feasible in any given situation, agents recommended for the treatment of febrile neutropenia should be constantly monitored and used as evenly as possible to control antibiotic resistance.

Some groups have performed antibiotic rotation trials in febrile neutropenic patients with hematological malignancies [16]–[19]; however, few cycling protocols have been designed with a regimen that rotates recommended agents for febrile neutropenia monotherapy without quinolone prophylaxis. No study had definitely shown a significant reduction in the incidence of particular antibiotic-resistant bacteria among febrile neutropenic patients after antibiotic cycling. Our trial suggests a positive impact of antibiotic rotation on the control of antimicrobial resistance. In this study, the use of carbapenems as an initial treatment for febrile neutropenia did not induce the emergence of either *Stenotrophomonas maltophilia* or metallo-β-lactamase-producing bacteria. Treatment with all the first line antibiotics recommended for febrile neutropenia was well tolerated in our trial. The strategy of “antibiotic heterogeneity” would be useful for the control of antimicrobial resistance, particularly in the treatment of febrile neutropenia, in which long-term administration of broad-spectrum antibiotics is often needed.
